# Low-Carbon Transition Models of High Carbon Supply Chains under the Mixed Carbon Cap-and-Trade and Carbon Tax Policy in the Carbon Neutrality Era

**DOI:** 10.3390/ijerph191811150

**Published:** 2022-09-06

**Authors:** Liang Shen, Fei Lin, T. C. E. Cheng

**Affiliations:** 1 School of Public Finance and Taxation, Shandong University of Finance and Economics, Jinan 250014, China; 2 PolyU Business School, The Hong Kong Polytechnic University, Hung Hom, Kowloon, Hong Kong 999077, Hong Kong

**Keywords:** carbon transition, mixed carbon policy, carbon cap-and-trade, carbon tax, sustainable supply chain

## Abstract

To achieve the goals of carbon peak and carbon neutrality, the low-carbon transformation (LCT) of high-carbon firms is inevitable. We construct game models of a supply chain with different dominant types under a mixed carbon policy that embraces carbon cap-and-trade and carbon tax. Solving each dominant model, we derive the effective area and optimal threshold of the mixed carbon policy to guide LCT. We find that the selling price, market demand, and profit of the supply chain system are equal in different dominant models due to the mixed carbon policy, but when a company dominates the supply chain, its profit is higher than when it is a subordinate. In addition, the high-carbon manufacturers (HCM) will pursue LCT only when the sum of the carbon tax rates and carbon trading prices is within a certain threshold, and the subordinate HCM are more likely to be driven to pursue LCT. Therefore, the government should adopt a differentiated hybrid carbon policy, setting a high (low) carbon tax rate for the HCM in a dominant (subordinate) position.

## 1. Introduction

With the growth of the low-carbon economy, the low-carbon transformation (LCT) of the high-carbon firms has attracted constant attention [1]. The LCT explains how the company incorporates low-carbon and environmental protection principles into its production operations. Naturally, this is also inseparable from the government’s carbon policy, which provides advice and assistance for LCT [2]. Net Zero Tracker run by the Energy & Climate Intelligence Unit (ECIU) shows that 136 nations and regions have made a commitment to achieving carbon neutrality, and that governments are starting to put strategic plans for a low-carbon transition in their supply chains into practice [3]. For example, the EU region has introduced the Fitfor 55 package, which aims to reduce carbon emissions by 55% by the end of 2030 compared to 1990. Utilizing the Carbon Border Adjustment Mechanism (CBAM), which makes imported goods responsible for the cost of carbon emissions, the global supply chain is encouraged to switch to a low-carbon economy [4]; The Build Back Better Act (BBBA) was passed in the USA to put the new energy deferred investment tax credits (ITCs) and production tax credits into effect (PTCs); In the “2022 China Low Carbon Supply Chain & Logistics Innovation Development Report”, China establishes various carbon policies for various industries and businesses [5]. However, 10% of Chinese firms still fall short of the emission reduction goal, according to the “Investigation Report on Energy Conservation and Emission Reduction of Chinese Enterprises during the 13th Five-Year Plan” [6]. Accordingly, it is important to research how carbon policy affects businesses that are transitioning from high- to low-carbon operations.

Most of the existing studies on the LCT of supply chains only consider a single carbon policy focusing on firms investing in low-carbon R&D [7,8,9,10]. However, we pay special attention to the high-carbon manufacturers (HCMs). HCMs refer to firms with intensive energy consumption and high environmental impact, covering industries such as electric power, papermaking, and paper product firms, textile firms, and oil and gas exploration. Their production model typically follows the traditional approach characterized with high cost, large scale, low efficiency, and low technology. Although LCT is a voluntary action for firms without governmental enforcement in many developing counties [11], but HCMs often have a low willingness to invest in the low-carbon technologies. There are two reasons for this. On the one hand, green low-carbon production requires large investments, takes long time, and involves high risks. Business firms’ economic interests often conflict with society’s ecological interests [12]. For example, TianYanCha [13] reported that, in 2021, among 1012 papermaking firms in Jinan, China, only six were engaged in LCT. On the other hand, HCMs lack funds for LCT and are unable to obtain financing support due to their limited strengths and low credit ratings [14]. Unlike low-carbon firms that also must make carbon emission reduction decisions [15,16], the HCMs in the article do not make carbon emission reduction investment decisions and the decision is only pricing. Furthermore, the difficulty in achieving carbon reduction lies in such HCMs. Thus, how to guide such HCMs for LCT through governmental policies requires a careful study.

Due to different market powers, HCMs may play two possible roles in supply chains. Energy-related HCMs often have great strengths and therefore dominate supply chains, which is common in developing countries, such as China and India [17]. Other HCMs may become subordinate elements of the supply chain as technology and markets evolve, and retailers tend to be market leaders in the supply chain, especially in developed countries. In this study we explore how to effectively guide the two types of high-carbon supply chains (HCSCs) for LCT, with a HCM as a market leader or a follower. In addition, different from previous studies, we consider a mixed carbon policy that embraces both the carbon tax and carbon cap-and-trade. The effect of implementing a single carbon policy is often not ideal [18]. Carbon cap-and-trade is a quantitative carbon pricing mechanism, in which the carbon price fluctuates due to changes in the demand for carbon emission rights. This means an uncertainty for HCMs’ expected return of investing in low-carbon technologies and thus hurts their investment motivation for LCT. In theory, the carbon tax policy will have a great impact on energy-intensive industries. In practice, however, firms often seek exemptions to maintain their competitiveness, so governments cannot achieve the expected emissions reduction. For example, in Denmark, Sweden, and the Netherlands, the carbon taxes have only a slight inhibitory effect on carbon emissions. The actual carbon emissions have not significantly dropped because of excessive exemptions for the energy-intensive industries [18]. Therefore, a single carbon policy can encourage general firms for LCT, but is less effective in motivating HCMs to pursue LCT. The mixed carbon policy proposed in this study can overcome the shortcomings of a single carbon policy. The carbon tax is essentially a fixed carbon price, so HCMs have stable expected returns on their low-carbon technology investment, rendering them willing to conduct low-carbon technology research. On the other hand, carbon cap-and-trade can overcome the shortcomings of carbon tax in restraining total carbon emissions. To achieve the emissions reduction targets, some countries have implemented both the carbon taxes and carbon cap-and-trade policies. For example, the EU announced that electric car dealers must join carbon cap-and-trade in addition to paying carbon taxes after achieving carbon neutrality in 2050. Fifteen countries and regions, including Canada, France, and the United Kingdom, have implemented both carbon cap-and-trade and carbon tax policies. Most of these countries have achieved carbon neutrality [19]. This shows that the mixed carbon policy has a better guiding effect on HCMs for LCT than a single carbon policy.

Specifically, the above observations raise to the following research questions: How does the mixed carbon policy affect pricing, HCSC’s profits, and HCM’s LCT? What mixed carbon policy does the government tend to adopt to guide the different roles of HCM’s LCT?

To answer the above questions, we will construct models of HCSCs under the mixed carbon policy. Our main contributions are as follows:

(1) Unlike previous studies that have focused on firms that actively conduct low-carbon R&D [20,21], we consider HCMs that do not invest in low-carbon R&D and build game models of HCSCs in different dominant modes, with the HCM as a market leader or a follower, which has further improved the application of low-carbon supply chain theory.
(2) Due to the limited guidance provided by a single carbon policy for HCM’s LCT, we consider the mixed carbon policy to identify the effects of the carbon trading price and carbon tax rate on pricing and HCSCs’ profits. We then focus on the LCT decisions of HCM under different dominant models of HCSCs and provide practical reference value for firms to make reasonable decisions.
(3) We find that the carbon trading price and carbon tax rate can direct the HCM’s LCT only if they are within the effective range, which is different from the conclusion that LCT for manufacturers can only be facilitated if the carbon tax rate is greater than a boundary condition [22]. In addition, under the mixed carbon policy, the subordinate HCM in the supply chain is more likely to be guided by LCT. Meanwhile, carbon cap-and-trade has the same impact on the different types of HCMs’ LCT, but the carbon tax has a greater impact on the subordinate HCM’s LCT.
(4) Finally, we present a differentiated mixed carbon policy for the government to guide HCMs’ LCT, which is different from the existing literature [23,24,25]. Specifically, the mixed carbon policy should not exceed a certain threshold for the manufacturer-led supply chain (M-SC); the mixed carbon policy should not exceed another threshold for the retailer-led supply chain (R-SC). Moreover, the government should set a higher carbon tax rate for the HCM with a greater market power and set a lower carbon tax rate for a weaker HCM when the carbon price is established in the carbon trading market.

We organize the rest of the paper as follows: Section 2 reviews the related literature. In Section 3 we introduce the models and discuss the assumptions. In Section 4 we analyze the HCSCs under different dominant models. In Section 5 we present the numerical studies to generate insights from the analytical findings. Section 6 concludes the paper and suggests topics for future research. We present the proofs of all the results in the Appendices.

## 2. Literature Review

Our research is related to three research streams, namely impact of carbon emissions reduction policies on the supply chain, the low-carbon decisions of supply chain, and the LCT of HCM.

### 2.1. Impact of Carbon Emissions Reduction Policies on the Supply Chain

Common carbon emission policies include carbon taxes, carbon cap-and-trade, and low-carbon subsidies. At present, most related research concentrates on the impact of different carbon emission policies on supply chain decisions, such as production, pricing, transportation, inventory, etc., and the resulting performance. In terms of carbon tax, Herber et al. [26] stated that the goal of a carbon tax, which is a type of shelter tax, is to internalize the external consequences of business carbon emissions through effective short-term carbon emission reduction; Yenipazarli, et al. [27] use the leader-follower Stackelberg game model to illustrate how an emissions tax can be developed to achieve the win-win economic, environmental, and social benefits inherent in remanufacturing; Fang et al. [28] found that the carbon tariff does not necessarily reduce global carbon emissions. In terms of carbon cap-and-trade, Qiu et al. [29] considered the pollution production-routing problem under carbon cap-and-trade and explored the carbon trading price’ impacts on the supply chain’s choice of the transport route. Chai et al. [30] discussed the effect of carbon cap-and-trade on a remanufacturing supply chain’s profit and recycling activities. Zhang et al. [31] developed an evolutionary game model between the government and a manufacturer to analyze the government policy’s impact on the carbon trading market. In terms of low-carbon subsidy, scholars usually analyze them in comparison with other policies, such as Cao et al. [32] found that the carbon emission reduction level is positively related to the carbon trading price and is not related to the low-carbon subsidy, by discussing the impacts of carbon cap-and-trade and the low-carbon subsidy on supply chain performance and the carbon emissions reduction level. Guo et al. [33] compared the social welfare under carbon tax and carbon subsidy and concluded that charging a carbon tax on a high carbon emitter is better than a carbon subsidy. Chen et al. [34] also make the point that carbon taxes levied by the government are more successful than subsidies at promoting low-carbon manufacturing. Accordingly, there are also comparative analyses of cap-and-trade and carbon taxes, such as Zhang et al. [35] explored the effects of cap-and-trade on carbon emissions and profits of a supply chain and also compared carbon cap-and-trade with carbon tax, illustrating the effectiveness of the cap-and-trade policy; Li et al. [36] conducted empirical research on comparing the effects of the carbon tax and carbon cap-and-trade on a coal supply chain network through an optimization framework.

Most of the above literature only considers a single carbon policy or compares the effects of different carbon policies. For mixed carbon policy, scholars often study carbon subsidies in combination with the other two policies [37,38,39]. Few scholars study the hybrid policy combination of carbon trading and carbon tax, which are becoming important lately [40]. Plambeck et al. [41] examined the impacts of carbon tax and carbon cap-and-trade on a material manufacturer’s profit, finding that the carbon tax or carbon cap-and-trade would reduce the manufacturer’s profit. Jin et al. [42] studied the impacts of carbon cap-and-trade and the carbon tax on a large retailer’s sales and logistics network design. Drake et al. [43] studied the impacts of carbon tax and carbon cap-and-trade on a firm’s technology choices. The mixed carbon policy can offset the shortcomings of a single carbon policy. However, there are not enough pertinent studies on the hybrid policy of carbon trading and carbon tax based on HCSC. Therefore, we study the impacts of the mixed policy embracing the carbon tax and carbon cap-and-trade on the HCSC.

### 2.2. Low-Carbon Decision-Making in the Supply Chain

The low-carbon decision of the supply chain plays a vital role in achieving the goal of net-zero carbon emissions. As research into the ability of product design to mitigate environmental impacts and improve efficiency has intensified [44], supply chain mitigation strategies have become a focus of scholarly research. For example, Ji et al. [45] considered the consumer’s low-carbon preference and analyzed the supply chain’s single emissions reduction and joint emissions reduction strategies. They found that the joint emissions reduction strategy is more beneficial to the manufacturer and retailer. Yuan et al. [46] constructed a multi-stage emissions reduction model, adopting discrete-time optimal control theory to derive the optimal emissions reduction strategy for each stage. Gopalakrishnan et al. [47] analyzed the supply chain’s low-carbon decisions of joint production under a carbon tax and internal carbon pricing. Zhou et al. [37] studied the competing manufacturers’ optimal joint pricing under the carbon tax and carbon subsidy and provided help for firms in pricing under supply chain competition. Liu et al. [48] compared the consumer surplus of the manufacturer’s emissions reduction model, the retailer’s emissions reduction model, and the joint emissions reduction model. They found that the joint emissions reduction model’s emissions reduction level is the highest. Zhang et al. [49] developed an emissions reduction dynamic model in a dual-channel supply chain to derive the consumers’ optimal low-carbon decisions under low-carbon preference. Applying game models of a supply chain consisting of a manufacturer and a retailer to study the implications of low-carbon investment and green marketing, Xia et al. [50] designed a contract for the supply chain’s coordination.

The above literature considers the decisions of the supply chain that is willing to choose low-carbon production. However, HCM do not actively choose LCT due to the carbon risks [51], high cost [52], and long time to realize low-carbon technology improvement [53]. It is urgent to achieve the goals of carbon peak and carbon neutrality. Therefore, we build the HCSC model to explore how to guide the HCM that does not actively engage in low-carbon production for LCT.

### 2.3. LCT of the HCM

Due to the low willingness of HCMs to participate in LCT, it is difficult to address the issue of motivating HCMs for LCT. Most related literature concerns evaluating the current’ carbon level and effect of situation. Zhou et al. [51] established a carbon risk evaluation system for the pursuit of LCT in heavy polluting industries. They found that the evaluation system can predict the carbon risk level of LCT. Wen et al. [54] used a system dynamics model to predict and compare heavy industries’ carbon emissions in Baoding, China, and made suggestions for achieving LCT. Based on Caohejing High-Tech Industrial Park in China, Huang et al. [55] analyzed the construction industry’s energy-saving ways, meanwhile, evaluated how the Caohejing Industrial Park reduced greenhouse gas emissions. Yu et al. [56] discussed and compared the LCT status of state-owned firms in China’s high-carbon industries, private firms, and non-high-carbon firms. They found that the LCT effect of high-carbon state-owned firms is not obvious. Based on scenario analysis, Wang et al. [57] compared the LCT potential of three tobacco companies through process optimization and product structure upgrades, and finally provided recommendations. By empirical research, Li [58] discovered that 30 per cent of power firms in China have high LCT efficiency. Zhang et al. [52] proposed a carbon value flow analysis-based carbon emissions cost calculation method. They provided an effective tool for high-carbon firms to carry out LCT. Dai et al. [59] used the Super-SBM model to discuss the thermal power firms’ dynamic performance variations under carbon emissions constraints in the past ten years. Zhang et al. [60] constructed a carbon budget system for high-carbon firms. They found that a carbon budget system can dynamically adjust the carbon emissions reduction level according to interactive control.

Most of the existing studies concentrate on evaluating the LCT effect of high-carbon firms and have not proposed effective methods to promote LCT. Different from the above literature, we study the effective range and optimal threshold for the HCM’s LCT under different dominant models of HCSCs. We also provide a theoretical reference for the HCM’s LCT.

## 3. Model Description and Assumptions

**Figure 1:**
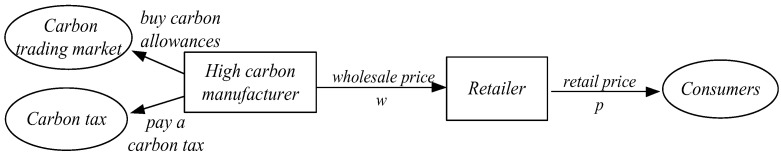
Model structure of the HCSC.

Deloitte [61] surveyed 76 Chinese firms, 37% from the energy and industrial sectors, 27% from the consumer goods sector, and 20% from the financial sector. They found that more than half of the firms believe that promoting efforts against climate change should be the government’s main responsibility, and they are reluctant to invest in LCT Xia [62]. Therefore, taking the current situation of HCMs in China as an example, we consider a supply chain consisting of a retailer and an HCM. The high-carbon manufacturer, as the manufacturer of the product, sells the product to the retailer at a wholesale price, and then sells it to the consumer at the going rate. Figure 1 shows the operation structure of HCSC, which covers the entire manufacturing to sales process.

Due to financial constraints, the HCM does not invest in emission reduction technology innovations. We investigate a mixed carbon policy, including both carbon cap-and-trade and carbon tax, to regulate the production of HCM. The manufacturer sells high-carbon products to the retailer at the wholesale price w and the retailer then resells the products to consumers at the retail price p. The market is featured by a demand function q=α−βp, where q is the market demand, α>0 is the potential market size, and β>0 is the elasticity coefficient of the demand to retail price. The manufacturer, who produces a high-carbon product with the unit production cost c and unit carbon emissions e, is subject to a carbon cap G, which is obtained through benchmark carbon allowance allocation [50]. The benchmark carbon allowance allocation is to issue a carbon cap based on the average carbon emissions of the industry. When the total emissions exceed the carbon cap, the manufacturer must purchase carbon quote with the price of t from the carbon trading market to cover the difference [63,64]. In addition, the manufacturer must pay a carbon tax at the rate of k for the carbon emissions produced. Among them, the carbon trading price is determined by the market and there is volatility, while the carbon tax is set by the government and plays a role in stabilizing carbon prices. Therefore, the carbon tax and carbon trading price inevitably affect the HCSC but are not affected by the HCSC. To better analyze the model and summarize the conclusions, they are set as exogenous variables.

To facilitate model analysis, we adopt the following assumptions:

**Assumption** **1** **(A1).** *The HCM has the characteristics of high carbon emissions and low willingness for LCT* [65]*, so their carbon emissions often exceed the carbon cap allocated by the government, i.e., eq−G>0.*

**Assumption** **2** **(A2).** *To ensure that the optimal decision is positive, we assume that the parameters meet the condition of *$k+t<\frac{e\left(\alpha -\beta c\right)-8G}{e^{2}\beta }$.

*The HCM’s profit function* $\pi_{m}$ *is*

(1) $$\pi_{m}=\left(w-c\right)q-t\left(eq-G\right)-keq$$

*the retailer’s profit function* $\pi_{r}$ *is*

(2) $$\pi_{r}=\left(p-w\right)q$$

*and the HCSC’s profit function* π *is*

(3) $$\pi =\pi_{m}+\pi_{r}$$

## 4. Model Construction and Analysis

The high-carbon manufacturer-led and retailer-led games in a high-carbon supply chain are modeled in this chapter, and the best solution to each model is presented separately. In addition, we compare the sales price, market demand, and profit of the two dominant models under a mixed carbon policy. Moreover, we provide the optimal threshold and operational area for the transition of the supply chain to a low-carbon economy.

### 4.1. Manufacturer-Led Supply Chain (M-SC) Model

When HCM is powerful (such as Baosteel in Shanghai, China, Mittal Steel in the Netherlands, and Posco in South Korea), the HCM and retailer constitute a Stackelberg game relationship with the HCM as the leader and the retailer as the subordinate. For the leader, the HCM first decides the wholesale price w. Then the retailer decides the retail price p in response to the HCM’s decision.

The HCM’s decision problem is

(4) $$\begin{array}{c} \underset{w}{Max}\;\pi_{m}=\left(w-c\right)q-t\left(eq-G\right)-keq\\ s.t.\;eq-G\geq 0 \end{array}$$

The retailer’s decision problem is

(5) $$\underset{p}{max\;}\pi_{r}=\left(p-w\right)q$$

By a backward induction, we see that there is a maximum value of $\pi_{r}$ at p=α+βw/2β. Substituting p into $\pi_{m}$, we find that $\pi_{m}$ is a concave function in w. Thus, we derive the optimal solution using the Karush–Kuhn–Tucker (KKT) condition as shown in Table 1 (Please refer to Appendix A for the proof).

### 4.2. Retailer-Led Supply Chain (R-SC) Model

When the HCM has a weak market power and the retailer has more influence in the supply chain, they form an R-SC and play a Stackelberg game with the retailer as the leader and the HCM as the follower. The retailer first determines the target marginal δ, where δ=p−w. Then, the HCM decides the wholesale price w in response to the retailer’s decision. The market demand for the product is

(6) q=α−β(δ+w)

The HCM’s decision problem is

(7) $$\begin{array}{c} \underset{w}{max}\;\pi_{m}=\left(w-c\right)q-t\left(eq-G\right)-keq\\ \;s.t.eq-G\geq 0 \end{array}$$

The retailer’s decision problem is

(8) $$\underset{\delta }{max}\;\pi_{r}=\delta q=\delta \left(\alpha -\beta \left(\delta +w\right)\right)$$

$\pi_{m}$ is a concave function in $w^{M*}$. We first solve (7) and obtain$\;w=\frac{\beta c+\beta ek+\beta et+\alpha -\beta \delta }{2\beta }$ using the KKT condition. Substituting w and q into$\;\pi_{r}$, we find that$\;\frac{\partial^{2}\pi_{r}}{\partial \delta^{2}}=-\beta <0$. By setting$\;\frac{\partial \pi_{r}}{\partial \delta }=0$, we obtain $\delta^{R*}=\frac{a-\beta c-\beta ek-\beta et}{2\beta }$. Therefore, we obtain the optimal decision as shown in Table 1 (Please refer to Appendix B for the proof).

### 4.3. Model Comparison Analysis

**Proposition** **1.** $p^{M*}$, $p^{R*}$ *,* $w^{M*}$ *, and* $w^{R*}$ are positively related to t. $q^{M*}$, $q^{R*}$, $\pi_{m}^{M*}$, $\pi_{m}^{R*}$, $\pi_{r}^{M*}$, and $\pi_{r}^{R*}$are negatively related to t.

**Proof** **of** **Proposition** **1.** Please refer to the Appendix C for details. □

From Proposition 1, we find that the carbon trading price affects the pricing and profits of both the retailer and HCM. A higher carbon trading price will increase the wholesale price and retail price, while reducing the market demand and the profits of HCSC members. In order to cover a higher carbon emissions cost, the HCM increases the wholesale price to transfer the cost to the retailer and finally to the final consumer. As a result, consumers share part of the cost of carbon emission, which leads to a decline in product demand and HCSC members’ profits.

**Proposition** **2.** $p^{M*}$, $p^{R*}$, $w^{M*}$, and $w^{R*}$are positively related to k. $q^{M*}$, $q^{R*}$, $\pi_{m}^{M*}$, $\pi_{m}^{R*}$, $\pi_{r}^{M*}$, and $\pi_{r}^{R*}$are negatively related to k.

**Proof** **of** **Proposition** **2.** The proof is the same as that for Proposition 1. □

From Proposition 2, we can see how the carbon tax rate affects the pricing and the profits of HCSC members. For different types of HCSCs, the carbon tax will increase the wholesale and retail prices, and reduce the market demand and HCSC members’ profits. This is because the HCM bears the additional cost of carbon emissions due to the carbon tax. Similar to Proposition 1, the profits of HCM and retailer are low in the long run. The carbon tax is a specific tax imposed by the government. The HCM can only reduce the cost of carbon emissions by reducing the carbon content of products. Therefore, the carbon tax can guide the HCM’s LCT to realize a low-carbon economy. For example, after Sweden implemented the carbon tax, the proportion of fossil fuel in industrial energy consumption was reduced to 30% [66]. Moreover, the proportion of the energy costs in the total costs has been declining, and the energy structure has been improved. Propositions 1 and 2 indicate that if the HCM does not adopt low-carbon technologies, they will face double pressure from the market and government, and profits will be low in the long run. Under such circumstances, the HCM faces two choices, one is bankruptcy, and the other is to actively adjust the production structure and adopt low-carbon technology.

**Proposition** **3.** (1) $\;w^{M*}>w^{R*}$, $\;p^{M*}=p^{R*}$, $\;\pi_{m}^{M*}>\pi_{m}^{R*}$, $\;\pi_{r}^{R*}>\pi_{r}^{M*}$, and $\;\pi^{M*}=\pi^{R*}$and (2) $\;\left|\frac{\partial \pi_{m}^{M*}}{\partial t}\right|>\left|\frac{\partial \pi_{m}^{R*}}{\partial t}\right|$, $\;\left|\frac{\partial \pi_{m}^{M*}}{\partial k}\right|>\left|\frac{\partial \pi_{m}^{R*}}{\partial k}\right|$, $\left|\frac{\partial \pi_{r}^{R*}}{\partial t}\right|>\left|\frac{\partial \pi_{r}^{M*}}{\partial t}\right|$, $\;and\;\left|\frac{\partial \pi_{r}^{R*}}{\partial k}\right|>\left|\frac{\partial \pi_{r}^{M*}}{\partial k}\right|$.

**Proof** **of** **Proposition** **3.** Please refer to the Appendix D for details. □

From Proposition 3, we can see that the wholesale price and the HCM’s profit in the HCSC are higher than those in R-SC. The retailer’s profit in M-SC is lower than that in R-SC. However, under both models, the sales price, market demands, and the HCSCs’ profits are the same. This is because, under the mixed carbon policy, the HCSCs face the same production environment and the market demand for high-carbon products is the same. Therefore, the market sales volume and the retail price are the same, and the profits of HCSCs are the same, too. However, an HCM with stronger market power enjoys more profit by increasing the wholesale price, while a weaker HCM has lower profit. In addition, the mixed carbon policy has a greater impact on the stronger HCM’s (retailer)’s profit than the weaker HCM (retailer), i.e., $\left|\frac{\partial \pi_{m}^{M*}}{\partial t}\right|>\left|\frac{\partial \pi_{m}^{R*}}{\partial t}\right|$ and $\left|\frac{\partial \pi_{m}^{M*}}{\partial k}\right|>\left|\frac{\partial \pi_{m}^{R*}}{\partial k}\right|$ ($\;\left|\frac{\partial \pi_{r}^{R*}}{\partial t}\right|>\left|\frac{\partial \pi_{r}^{M*}}{\partial t}\right|$ and $\left|\frac{\partial \pi_{r}^{R*}}{\partial k}\right|>\left|\frac{\partial \pi_{r}^{M*}}{\partial k}\right|$).

Let C be the opportunity cost of the HCM and $F=\pi_{m}^{i*}-C,\;\left(i=M,R\right)$, i.e., the difference between the HCM’s profit and the opportunity cost. Opportunity cost refers to the highest income obtained by giving up using the same production factors for other purposes [67]. When F>0, the HCM’s profit is greater than the opportunity cost and the HCM will produce normally. Otherwise, the HCM will stop production and close down when F<0. Therefore, we obtain the following result.

**Proposition** **4.** *Under the mixed carbon policy,*

(1) *the M-SC’s LCT interval I satisfies $h^{M}\left(k,t\right)\leq 0$ when$\;h^{M}\left(k,t\right)>0$, and the HCM is forced to stop production. The HCM’s transition threshold A meets$\;h^{M}\left(k,t\right)=0$;*
(2) *the R-SC’s LCT interval II satisfies $h^{R}\left(k,t\right)\leq 0$ when $h^{R}\left(k,t\right)>0$, and the HCM is forced to stop production. The HCM’s transition threshold B satisfies $h^{R}\left(k,t\right)=0$;*

*Where $h^{R}\left(k,t\right)=t+k+\frac{4{\left(\beta e^{2}Gk+\beta Ce^{2}-\alpha eG+\beta ceG+4G^{2}\right)}^{\frac{1}{2}}}{\beta e^{2}}-\frac{\alpha e-\beta ce-8G}{\beta e^{2}}$, and $h^{M}\left(k,t\right)=t+k+\frac{2\sqrt{2}{\left(\beta e^{2}Gk+\beta Ce^{2}-\alpha eG+\beta ceG+2G^{2}\right)}^{\frac{1}{2}}}{\beta e^{2}}-\frac{\alpha e-\beta ce-4G}{\beta e^{2}}$.*

**Proof** **of** **Proposition** **4.** Please refer to the Appendix E for details. □

**Figure 2:**
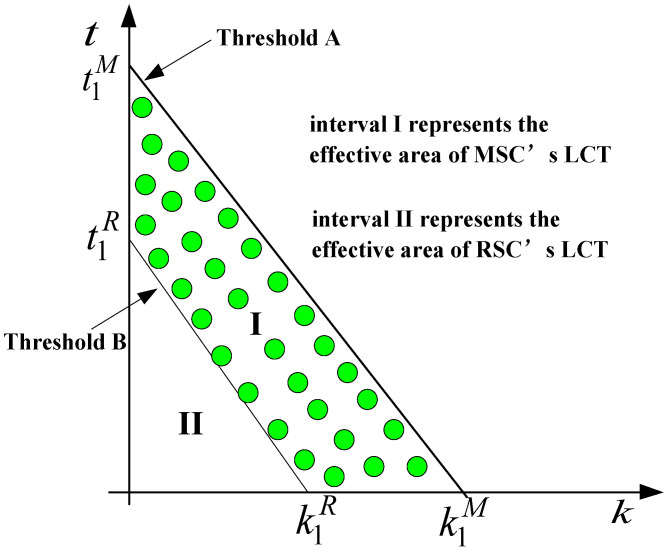
The HCM’s LCT interval.

We draw the analysis chart of the HCM’s LCT interval in Figure 2. In Figure 2, $t_{1}^{M}=\frac{\alpha e-\beta ce-4G-2\sqrt{2}{\left(\beta Ce^{2}-\alpha eG+\beta ceG+2G^{2}\right)}^{\frac{1}{2}}}{\beta e^{2}}$, $t_{1}^{R}=\frac{\alpha e-\beta ce-8G-4{\left(\beta Ce^{2}-\alpha eG+\beta ceG+4G^{2}\right)}^{\frac{1}{2}}}{\beta e^{2}}$, $k_{1}^{M}=\frac{\alpha -\beta c-\sqrt{8\beta C}}{\beta e}$, and $k_{1}^{R}=\frac{\alpha -\beta c-\sqrt{16\beta C}}{\beta e}$.

Since the firm’s goal is to maximize profit, it will continue to produce only when its profit is higher than the opportunity cost. Otherwise, it will stop production and use the production factors for other purposes [68]. Under the mixed carbon policy, the HCM’s profit decreases as the carbon tax rate and carbon trading price increase, so it must adjust its industrial structure when its profit is higher than the opportunity cost, i.e., $h^{i}\left(k,t\right)<0\;\left(i=M,R\right)$. Otherwise, with increases in the carbon trading price and carbon tax rate, i.e., $h^{i}\left(k,t\right)>0$, the HCM’s profit will be less than the opportunity cost and it will stop production. When $h^{i}\left(k,t\right)=0$, the HCM’s profit is equal to the opportunity cost and it must stop production if it does not choose LCT. Thus, when the mixed carbon policy’s pricing is below a certain threshold, the HCM must choose LCT, which enables it to a relative competitive advantage and occupy the low-carbon product market. Otherwise, a higher price will force the HCM to exit the industry. From Proposition 4, we obtain the profit changes of different types of HCSCs under the mixed carbon policy in Table 2.

From Proposition 4, together with Figure 2 and Table 2, we see that the two dominant types of HCSCs’ LCT intervals are different. When the combination of the carbon tax rate and carbon trading price is below threshold A, i.e., $h^{M}\left(k,t\right)=0$, the HCM in M-SC should choose LCT. Otherwise, the HCM will stop production and the HCSC will be interrupted. When the combination of the carbon tax rate and carbon trading price are below the threshold B, i.e., $h^{R}\left(k,t\right)=0$, the HCM in R-SC should choose LCT. Otherwise, the HCM will stop production and the supply chain will be interrupted. If the government adopts a unified mixed carbon policy, and the combination of the carbon tax rate and carbon trading price is set at the lower threshold B, the HCM in R-SC can easily be guided to pursue LCT. However, the HCM’s LCT effect in M-SC is not obvious. If the combination of the carbon tax rate and carbon trading price is set at the higher threshold A, the HCM in M-SC will be guided to pursue LCT, but the HCM in R-SC will stop production. Thus, to ensure that all HCMs choose LCT, the government should adopt a differentiated mixed carbon policy. For M-SC, the government’s mixed carbon policy should be in interval I and the optimal setting is threshold A. For R-SC, the government’s mixed carbon policy should be in interval II and the optimal setting is threshold B.

**Proposition** **5.** *The HCM in R-SC is more likely to be led to pursue LCT under the mixed carbon policy, i.e., $h^{M}\left(k,t\right)<h^{R}\left(k,t\right)$.*

The mixed carbon policy can guide the HCM to choose LCT when it does not exceed a certain threshold and the LCT effect is best when the threshold is reached. In M-SC, the HCM’s LCT point is threshold A, which is $h^{M}\left(k,t\right)=0$. In R-SC, the HCM’s LCT point is threshold B, which is $h^{R}\left(k,t\right)=0$. $h^{M}\left(k,t\right)<h^{R}\left(k,t\right)$, so, a weak HCM in a subordinate position in the supply chain is more likely to be led to pursuing LCT.

**Proposition** **6.** *Under the mixed carbon policy, the carbon trading price has the same impacts on the HCM’s LCT in M-SC and R-SC, i.e., $\frac{\partial h^{M}\left(k,t\right)}{\partial t}=\frac{\partial h^{R}\left(k,t\right)}{\partial t}$. However, the carbon tax rate has a greater impact on the HCM’s LCT in R-SC than in the M-SC, i.e., $\frac{\partial h^{R}\left(k,t\right)}{\partial k}>\frac{\partial h^{M}\left(k,t\right)}{\partial k}$.*

Proposition 6 indicates that the impact of the carbon trading price on the LCT of all types of HCMs are the same. However, the impact of the carbon tax rate is related to the strength of HCM. The carbon tax rate has a greater impact on the weaker subordinate HCM’s LCT. As the weaker HCM obtains a smaller profit, this is not good for the weaker HCM. The government should set a differentiated carbon tax based on the’ strengths of HCMs. Specifically, the government should set a higher carbon tax rate for powerful energy HCMs and a relatively low carbon tax rate for the weaker HCMs.

In addition to the mixed carbon policy, we define the following two cases,

**Case** **1.** A single carbon tax policy, i.e., t=0,k>0.

**Case** **2.** A single carbon-cap-trade policy, i.e., t>0,k=0.

**Proposition** **7.** *The mixed carbon policy has always worked better than the single carbon policy. (1) Compared with a single carbon tax policy and single carbon-cap-trade policy, the HCM is more likely to be led to pursue LCT under the mixed carbon policy, i.e., *$h^{i}\left(k,t\right)>h^{i}\left(k,0\right)>h^{i}\left(0,t\right),i=M,R$;

*(2)*$\pi_{m}^{i*}\left(k,t\right)<\pi_{m}^{i*}\left(k,0\right)$,$\;\pi_{m}^{i*}\left(k,t\right)<\pi_{m}^{i*}\left(k,0\right)$,$\;\pi^{i*}\left(k,t\right)<\pi^{i*}\left(k,0\right)$;

*(3)*$\pi_{m}^{i*}\left(k,t\right)<\pi_{m}^{i*}\left(0,t\right)$,$\;\pi_{m}^{i*}\left(k,t\right)<\pi_{m}^{i*}\left(0,t\right)$,$\;\pi^{i*}\left(k,t\right)<\pi^{i*}\left(0,t\right)$.

**Proof** **of** **Proposition** **7.** The proof is the same as that for Proposition 4. □

From Proposition 7, we can see that the HCSC and its members’ profits under the mixed carbon policy are lower compared with the single carbon tax and carbon-cap-trade policy. Thus, the pressure to reduce carbon emissions is more urgent, which makes the willingness of HCM’s LCT more intense. On the other hand, compared with the single carbon tax and carbon trading policy, the LCT interval of carbon trading price and carbon tax under the mixed carbon policy is larger and more favorable to HCM’s LCT. In summary, the mixed carbon policy has always worked better than the single carbon policy.

## 5. Numerical Studies

To further analyze the different types of HCSCs’ LCT strategies under the mixed carbon policy, we refer to Sauré et al. [69], Plambeck et al. [41], David et al. [70], and Chen et al. [71], setting the parameters  c=7, e=8, α=380, β=0.9, G=150, and C=7500. Letting t and k be independent variables, where t∈(0,30) and  k∈(0,25), we draw the following figures.

**Figure 3:**
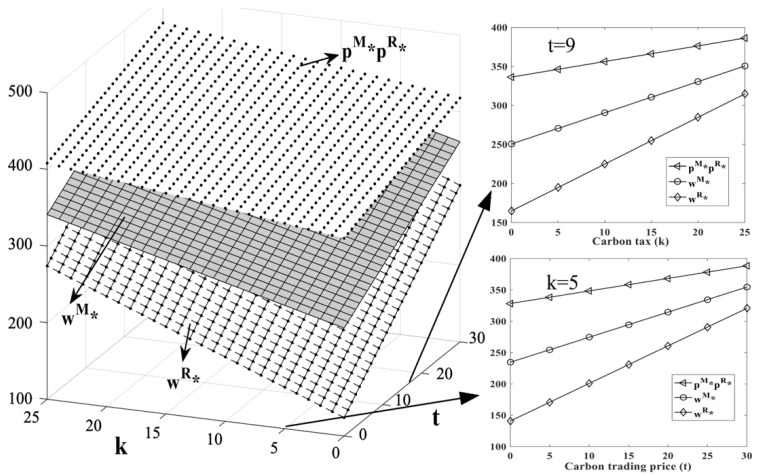
Changes of p and w regarding t and k.

**Figure 4:**
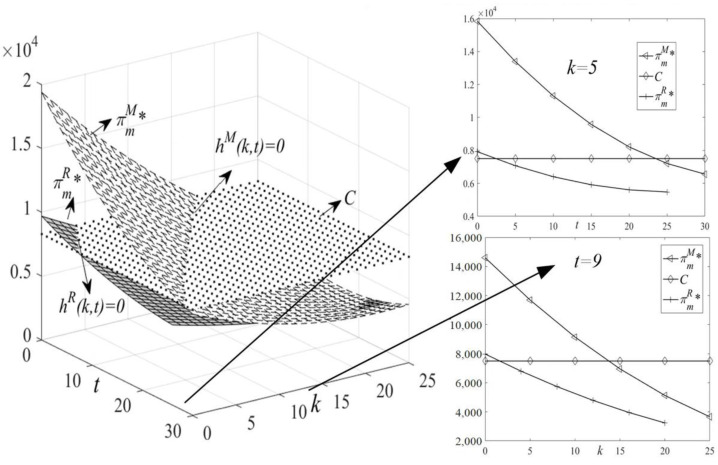
Changes of $\pi_{m}$ with t and k.

**Figure 5:**
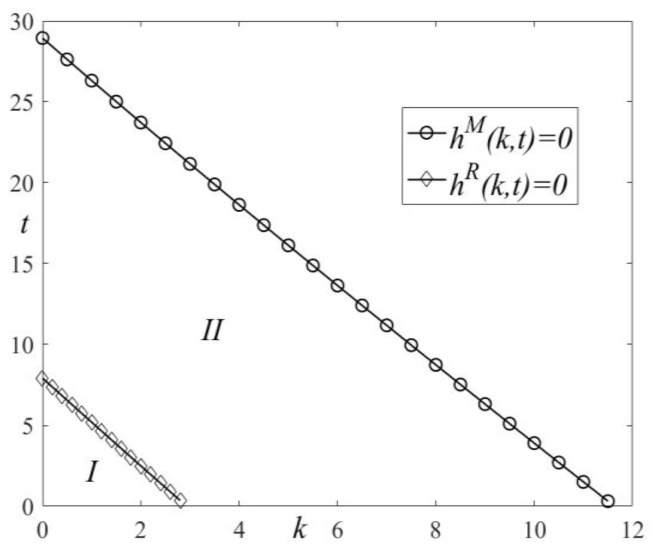
The LCT interval.

**Figure 6:**
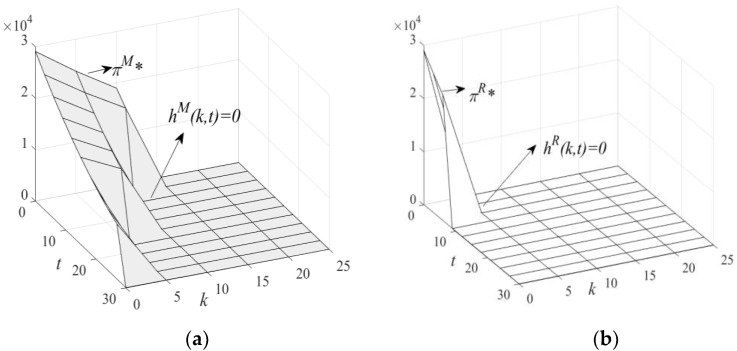
Changes of π in t and k, they should be listed as: (**a**) changes of $\pi^{M}$ in t and k; (**b**) changes of $\pi^{R}$ in t and k.

(1) Figure 3: HCSCs’ price changes with k and t.
(2) Figure 4: HCMs’ profit changes with k and t.
(3) Figure 5: LCT interval changes with k and t.
(4) Figure 6: HCSCs’ profit and LCT threshold changes with k and t.

In addition, we plot price and HCMs’ profit changes with the independent variables t and k in Figure 3 and Figure 4, when k=5 and t=9, respectively.

Figure 3 verifies Propositions 1 and 2. As the carbon trading price and carbon tax rate increase, the prices of retail and wholesale under different dominant models will increase. This is because the mixed carbon policy increases the cost of carbon emissions for the HCM. To cover the increased cost, the HCM will increase the wholesale price and transfer the cost to the retailer. To ensure that the marginal revenue remains unchanged, the retailer will increase the sales price to make the consumers bear part of the carbon emissions cost.

Figure 4 shows that taking a dominant position in the supply chain is conducive to obtaining more profit. In M-SC, the HCM’s profit is higher, while its profit in R-SC is severely compressed (Proposition 3). Moreover, distinct types of HCM’s profits decrease with the carbon trading price and carbon tax rate under the mixed carbon policy (Proposition 4). When $h^{M}\left(k,t\right)\leq 0$ ($h^{R}\left(k,t\right)\leq 0$), the HCM’s profit in M-SC (R-SC) is greater than the opportunity cost, so it can choose LCT. Otherwise, when $h^{M}\left(k,t\right)>0$ ($h^{R}\left(k,t\right)>0$), the HCM’s profit in M-SC (R-SC) is less than the opportunity cost, so it is forced to stop production.

From Figure 5 and Figure 6, we see that HCSCs’ profits satisfy $\pi^{M*}>0$ and $\pi^{R*}>0$ when $h^{i}\left(k,t\right)<0\left(i=M,R\right)$. With an increase in $h^{i}\left(k,t\right)$, $\pi^{M*}>0$ and $\pi^{R*}=0$ when $h^{R}\left(k,t\right)\geq 0$ and $h^{M}\left(k,t\right)<0$, respectively. Therefore, the condition of threshold *B*, i.e., $h^{R}\left(k,t\right)=0$, is met. At this time, the weaker HCM in R-SC must choose LCT; otherwise, it will go bankrupt. On the other hand, the stronger HCM can maintain its existing production and can also choose LCT. As the carbon tax rate and carbon trading price continue to increase, the condition of threshold A, i.e., $h^{M}\left(k,t\right)=0$, is also met. The HCM in M-SC must choose LCT; otherwise, it will go bankrupt. When $h^{R}\left(k,t\right)>0$ and $h^{M}\left(k,t\right)>0$, the supply chain members’ profits are $\pi^{M*}=0$ and $\pi^{R*}=0$. Therefore, the bearing capacity of HCM is also different because its strength and dominance are different in the HCSC. To guide LCT, the government should adopt a differentiated mixed carbon policy. For M-SC, the combination of the carbon tax rate and carbon trading price cannot be higher than threshold A and should be in interval I. For R-SC, the combination of the carbon tax rate and carbon trading price cannot be higher than threshold B and should be in interval II. Threshold B is met first, which further shows that the HCM in R-SC is more likely to be guided to pursue LCT (Proposition 5).

**Figure 7:**
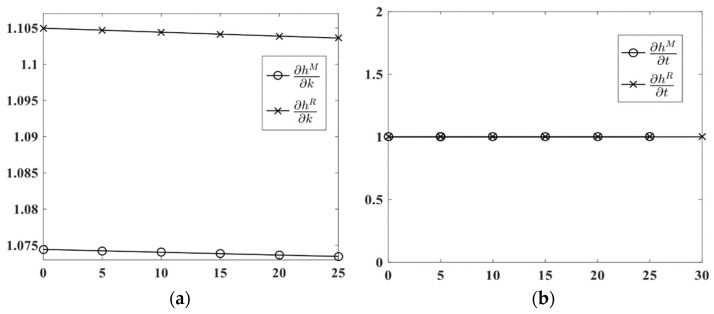
The impacts of t and k on LCT, they should be listed as: (**a**) The impact of k on LCT; (**b**) The impact of t on LCT.

Using the same set of parameters, i.e., c=7, e=8, α=380, β=0.9, G=150, and C=7500, and letting k be an independent variable, where k∈(0,25), we plot the LCT threshold’s change rate $\partial h^{i}\left(k,t\right)/\partial k\left(i=M,R\right)$ with k in Figure 7a. Letting t be an independent variable, where t∈(0,30), we plot the LCT threshold’s change rate $\partial h^{i}\left(k,t\right)/\partial t\left(i=M,R\right)$ with t in Figure 7b.

## 6. Conclusions

The LCT of HCM is imminent for achieving the goal of carbon neutrality. To examine the effects of the mixed carbon policy on the LCT of HCS, we construct game models of a supply chain consisting of an HCM and a retailer in different dominant modes, namely M-SC and R-SC. We derive the optimal decisions and LCT conditions of each dominant model. Then we compare and analyze two models, M-SC and R-SC, to find the effective interval and optimal threshold to guide the LCT of HCS under the mixed carbon policy. The conclusions are as follows:

(i) The dominant model is different, and the profits shared by the HCM and the retailer are also different under the mixed policy. We find that “the dominant firm has more profits”, which conforms to the findings of Yue et al. [72], Wang et al. [73], and Zhang et al. [74]. But the difference is that the sales prices, market demands, and supply chain profits are the same under the two dominant models due to the impact of the mixed carbon policy. Moreover, the carbon trading price and carbon tax rate are negatively correlated with HCSC and its members which is similar to Plambeck et al. [41], while being positively correlated with will raise the wholesale and retail prices under the mixed carbon policy, which is similar to Yenipazarli, et al. [27].
(ii) For firms, the LCT decisions of different types of HCM are as follows: For M-SC/R-SC, when the carbon tax rate and carbon trading price under the mixed carbon policy do not exceed threshold A (B), i.e., $h^{M}\left(k,t\right)=0$ ($h^{R}\left(k,t\right)=0$), the HCM should choose LCT, which is a more precise range of values than that given by wang et al. [22] under the single carbon tax policy. When threshold A or B is reached, the HCM must choose LCT. When threshold A or B is exceeded, the HCM should stop production.
(iii) For the government, we contend that the government should adopt a differentiated mixed carbon policy to promote LCT to different types of HCM, in contrast to Guo et al. [33], Chen et al. [34], and Zhang et al. [35] who support the implementation of a single carbon policy. For M-SC, the mixed carbon policy should be within the effective interval I and the LCT point should be threshold A. For R-SC, the mixed carbon policy should be in the effective interval II and the LCT point should be threshold B. Moreover, the government should set a higher carbon tax rate for the more powerful dominant HCM in M-SC, and a relatively lower carbon tax rate for the subordinate HCM in R-SC.

According to our research, the differentiated mixed carbon policy is effective in guiding different leading types of the LCT of HCM, which require actions by both government and business. The following managerial insights can be drawn and can be applied not only to the Chinese market but also to others.

First, the government can implement the following actions. On the one hand, because carbon cap-and-trade has the same impact on the HCM, and the carbon tax policy has a stronger regulation effect on the weaker HCM, the government should set the higher carbon tax rate for the more powerful HCM than the weaker. On the other hand, in order to speed up LCT, the government should implement the pilot work of differentiated mixed carbon policy to accumulate experience for the full implementation of the mixed policy. Second, the HCM must make appropriate production decisions based on the carbon trading price and carbon tax rate pricing, specifically, when it is below than a certain threshold, the HCM should choose LCT. Third, to achieves “win-win” cooperation, the retailer needs to actively support HCM’s LCT in areas such as internal financing, technology, marketing, and cross-shareholding strategic cooperation. HCM’s suspension of production will cause disruptions in the HCSC, which also results in the decline of the retailer’s profit. For example, the clothing brand PUMA adopts a prepayment plan to encourage its manufacturers to implement low-carbon production and promote the LCT of the supply chain [75].

Our research has certain limitations. We assume that the firms have low willingness for LCT. HCMs will invest in low-carbon technologies before pursuing LCT. In addition, HCMs are subject to financial constraints in pursuing LCT, resulting in insufficient transformation efforts. Considering these factors in future research, we can get more realistic research results. In addition, consumers’ low-carbon preferences play a role in market regulation of the LCT of supply chain. Combining this market factor with the government’s guiding policies to study the LCT of supply chain will be our next research direction.

## Figures and Tables

**Table 1:** Optimal decisions and profits of different models.

HCSC	The Optimal Decisions and Profits of HCSC Members
M-SC	$w^{M*}=\frac{\alpha +\beta c+\beta ek+\beta et}{2\beta }$, $q^{M*}=\frac{\alpha -\beta \left[c+e\left(k+t\right)\right]}{4}$, $p^{M*}=\frac{3\alpha +\beta \left[c+e\left(k+t\right)\right]}{4\beta }$, $\pi_{r}^{M*}=\frac{{\left\{\alpha -\beta \left[c+e\left(k+t\right)\right]\right\}}^{2}}{16\beta }$, $\pi_{m}^{M*}=\frac{{\left\{\alpha -\beta \left[c+e\left(k+t\right)\right]\right\}}^{2}}{8\beta }+Gt$, $\pi^{M*}=\frac{3{\left\{\alpha -\beta \left[c+e\left(k+t\right)\right]\right\}}^{2}}{16\beta }+Gt$.
R-SC	$w^{R*}=\frac{\alpha +3\beta c+3\beta ek+3\beta et}{4\beta }$, $q^{R*}=\frac{\alpha -\beta \left[c+e\left(k+t\right)\right]}{4}$, $p^{R*}=\frac{3\alpha +\beta \left[c+e\left(t+k\right)\right]}{4\beta }$, $\pi_{r}^{R*}=\frac{{\left\{\alpha -\beta \left[c+e\left(k+t\right)\right]\right\}}^{2}}{8\beta }$, $\pi_{m}^{R*}=\frac{{\left\{\alpha -\beta \left[c+e\left(k+t\right)\right]\right\}}^{2}}{16\beta }+Gt$, $\pi^{R*}=\frac{3{\left\{\alpha -\beta \left[c+e\left(k+t\right)\right]\right\}}^{2}}{16\beta }+Gt$.

$w^{M*}$ is the optimal solution of the wholesale price per unit product in the M-SC. $q^{M*}$ is the market demand under the optimal solution in the M-SC. $p^{M*}$ is the optimal solution of unit product retail price in the M-SC. $\pi_{r}^{M*}$, $\pi_{m}^{M*}$, and $\pi^{M*}$ are the optimal profits of the retailer, HCM and HCSC in the M-SC. Similarly, $w^{R*}$ is the optimal solution of the wholesale price per unit product in the R-SC. $q^{R*}$ is the market demand under the optimal solution in the R-SC. $p^{R*}$ is the optimal solution of unit product retail price in the R-SC. $\pi_{r}^{R*}$, $\pi_{m}^{R*}$, and $\pi^{R*}$ are the optimal profits of the retailer, HCM and HCSC in the R-SC.

**Table 2:** Profit changes of different types of HCSC.

Mixed Carbon Policy Threshold	M-SC	R-SC
$h^{i}\left(k,t\right)<0\left(i=M,R\right)$	$\pi^{M*}>0$	$\pi^{R*}>0$
$h^{R}\left(k,t\right)\geq 0,h^{M}\left(k,t\right)<0$	$\pi^{M*}>0$	$\pi^{R*}=0$
$h^{R}\left(k,t\right)>0,h^{M}\left(k,t\right)\geq 0$	$\pi^{M*}=0$	$\pi^{R*}=0$

## Data Availability

No application.

## Appendix A

**Proof of Table 1 (M-SC).** 

(A1)
$$p=\frac{\alpha +\beta w}{2\beta }\;$$

(A2)
q=α−βp

Substituting Equation (A1) into Equation (A2), we get

(A3) $$q=\frac{\alpha -\beta w}{2}$$

Substituting Equation (A3) into Equation (4), we get

(A4) $$\begin{array}{c} \underset{w}{max}\pi_{m}=\left(w-c\right)\left(\frac{\alpha -\beta w}{2}\right)-t\left\{\frac{e\alpha -e\beta w}{2}-G\right\}-ke\frac{\alpha -\beta w}{2}\\ s.t.e\frac{\alpha -\beta w}{2}-G\geq 0 \end{array}$$

From Equation (A4), the Lagrange function is as follows:

$$\begin{array}{c} L\left(w,\lambda \right)=-\left(w-c\right)\frac{\alpha -\beta w}{2}+t\left(e\frac{\alpha -\beta w}{2}-G\right)+ke\frac{\alpha -\beta w}{2}+\lambda \left(G-e\frac{\alpha -\beta w}{2}\right)\\ \begin{array}{cc} s.t. & \lambda \left(G-e\frac{\alpha -\beta w}{2}\right)=0\\ & \lambda \geq 0 \end{array} \end{array}$$

The optimal solution of the decision problem must satisfy the following conditions:

$$\begin{array}{c} L\left(w,\lambda \right)=-\left(w-c\right)\frac{\alpha -\beta w}{2}+t\left(e\frac{\alpha -\beta w}{2}-G\right)+ke\frac{\alpha -\beta w}{2}+\lambda \left(G-e\frac{\alpha -\beta w}{2}\right)\\ \begin{array}{cc} s.t. & \lambda \left(G-e\frac{\alpha -\beta w}{2}\right)=0\\ & \lambda \geq 0 \end{array} \end{array}$$

The optimal solution of the decision problem must satisfy the following conditions:

$$\begin{array}{cc} & \frac{\partial L}{\partial w}=\frac{\beta w-\alpha -\beta c-\beta et-\beta ek+\lambda \beta e}{2}=0\\ & \lambda \left(G-e\frac{\alpha -\beta w}{2}\right)=0\\ & \lambda \geq 0\\ & G-e\frac{\alpha -\beta w}{2}\leq 0 \end{array}\;$$

When  λ>0, we derive that

(A5) $$\{\begin{array}{c} \frac{\beta w-\alpha -\beta c-\beta et-\beta ek+\lambda \beta e}{2}=0\\ G-e\frac{\alpha -\beta w}{2}=0 \end{array}$$

Solving Equation (A5), we get

$$\lambda =\frac{\beta ce+\beta e^{2}k+2G+\beta e^{2}t}{\beta e^{2}}\;$$

and

$$w=\frac{\alpha e-2G}{\beta e}\;$$

In this case, the firm’s total carbon emissions are always equal to the free carbon cap, which does not match the actual situation. When  λ=0, we derive that

(A6) $$w^{M*}=\frac{\alpha +\beta c+\beta ek+\beta et}{2\beta }$$

Substituting Equation (A6) into Equations (A1) and (A3), we get

(A7) $$p^{M*}=\frac{3\alpha +\beta \left[c+e\left(k+t\right)\right]}{4\beta }$$

and

(A8) $$q^{M*}=\frac{\alpha -\beta \left[c+e\left(k+t\right)\right]}{4}$$

Substituting Equations (A6)–(A8) into Equations (4) and (5), we get

$$\pi_{m}^{M*}=\frac{{\left\{\alpha -\beta \left[c+e\left(k+t\right)\right]\right\}}^{2}}{8\beta }+Gt$$

and

$$\pi_{r}^{M*}=\frac{{\left\{\alpha -\beta \left[c+e\left(k+t\right)\right]\right\}}^{2}}{16\beta }\;$$

Then the supply chain’s profit is

$$\pi^{M*}=\frac{3{\left\{\alpha -\beta \left[c+e\left(k+t\right)\right]\right\}}^{2}}{16\beta }+Gt\;$$

□.

## Appendix B

**Proof of Table 1 (R-SC).**  Same as Appendix A we get Equation (A9).

(A9) $$w=\frac{\beta c+\beta ek+\beta et+\alpha -\beta \delta }{2\beta }$$

Substituting $\delta =\frac{a-\beta c-\beta ek-\beta et}{2\beta }$ into Equation (A8), we get

(A10) $$w^{R*}=\frac{\alpha +3\beta c+3\beta ek+3\beta et}{4\beta }$$

and

$$p^{R*}=w^{R*}+\delta =\frac{3\alpha +\beta \left[c+e\left(t+k\right)\right]}{4\beta }$$

Substituting δ and Equation (A10) into Equation (6), we get

(A11) $$q^{R*}=\frac{\alpha -\beta \left[c+e\left(k+t\right)\right]}{4}$$

Substituting δ, Equations (A10) and (A11) into Equations (7) and (8), we get

$$\pi_{r}^{R*}=\frac{{\left\{\alpha -\beta \left[c+e\left(k+t\right)\right]\right\}}^{2}}{8\beta }$$

and

$$\pi_{m}^{R*}=\frac{{\left\{\alpha -\beta \left[c+e\left(k+t\right)\right]\right\}}^{2}}{16\beta }+Gt$$

Then the supply chain’s profit is

$$\pi^{R*}=\frac{3{\left\{\alpha -\beta \left[c+e\left(k+t\right)\right]\right\}}^{2}}{16\beta }+Gt$$

□.

## Appendix C

**Proof of Proposition 1.**  Given that $\frac{\partial p^{M*}}{\partial t}=\frac{\beta }{4}>0$, we can similarly prove that $\frac{\partial p^{R*}}{\partial t}>0$, $\frac{\partial w^{M*}}{\partial t}>0$, $\frac{\partial w^{R*}}{\partial t}>0$, $\frac{\partial q^{M*}}{\partial t}<0$, and $\frac{\partial q^{R*}}{\partial t}<0$. Noting that $\frac{\partial \pi_{m}^{M*}}{\partial t}=\frac{-\alpha e+\left\{4G+\beta e\left[c+e\left(k+t\right)\right]\right\}}{4}<0$, we can similarly prove that $\frac{\partial \pi_{m}^{R*}}{\partial t}<0$, $\frac{\partial \pi_{r}^{M*}}{\partial t}<0$, and $\frac{\partial \pi_{r}^{R*}}{\partial t}<0$. □

## Appendix D

**Proof of Proposition 3.**  Given $\pi_{m}^{M*}=\frac{{\left\{\alpha -\beta \left[c+e\left(k+t\right)\right]\right\}}^{2}}{8\beta }+Gt\;$ and $\pi_{m}^{R*}=\frac{{\left\{\alpha -\beta \left[c+e\left(k+t\right)\right]\right\}}^{2}}{16\beta }+Gt$, we have $\pi_{m}^{M*}-\pi_{m}^{R*}>0$. Thus, $\pi_{m}^{M*}>\pi_{m}^{R*}$. We can similarly prove that $\pi_{r}^{R*}>\pi_{r}^{M*}$, $w^{M*}>w^{R*}$, $p^{M*}=p^{R*}$, and $\pi^{M*}=\pi^{R*}$. Given $\frac{\partial \pi_{m}^{M*}}{\partial t}=\frac{-\alpha e+\left\{4G+\beta e\left[c+e\left(k+t\right)\right]\right\}}{4}<0\;$ and $\frac{\partial \pi_{m}^{R*}}{\partial t}=\frac{-\alpha e+\left\{8G+\beta e\left[c+e\left(k+t\right)\right]\right\}}{8}<0$, we have $\frac{\partial \pi_{m}^{M*}}{\partial t}<\frac{\partial \pi_{m}^{R*}}{\partial t}<0$. Thus, $\left|\frac{\partial \pi_{m}^{M*}}{\partial t}\right|>\left|\frac{\partial \pi_{m}^{R*}}{\partial t}\right|$. We can similarly prove that $\left|\frac{\partial \pi_{m}^{M*}}{\partial k}\right|>\left|\frac{\partial \pi_{m}^{R*}}{\partial k}\right|$, $\left|\frac{\partial \pi_{r}^{R*}}{\partial t}\right|>\left|\frac{\partial \pi_{r}^{M*}}{\partial t}\right|$, and $\left|\frac{\partial \pi_{r}^{R*}}{\partial k}\right|>\left|\frac{\partial \pi_{r}^{M*}}{\partial k}\right|$. □

## Appendix E

**Proof of Proposition 4.**  Let $F=\pi_{m}^{M*}-C$, which represents the difference between the HCM’s profit and the opportunity cost. Letting $F\left(k,t\right)=\pi_{m}^{M*}-C=0$, we see that k and t satisfy the following condition

(A12) $$t+k\pm \frac{2\sqrt{2}{\left(\beta e^{2}Gk+\beta Ce^{2}-\alpha eG+\beta ceG+2G^{2}\right)}^{\frac{1}{2}}}{\beta e^{2}}=\frac{\alpha e-\beta ce-4G}{\beta e^{2}}$$

To ensure that the decision is meaningful, we have $k+t<\frac{\alpha e-\beta ce-8G}{\beta e^{2}}$. Therefore, $t+k-\frac{2\sqrt{2}{\left(\beta e^{2}Gk+\beta Ce^{2}-\alpha eG+\beta ceG+2G^{2}\right)}^{\frac{1}{2}}}{\beta e^{2}}=\frac{\alpha e-\beta ce-4G}{\beta e^{2}}$ is discarded. The boundary condition when the dominant HCM’s profit is equal to the opportunity cost is

$$t+k+\frac{2\sqrt{2}{\left(\beta e^{2}Gk+\beta Ce^{2}-\alpha eG+\beta ceG+2G^{2}\right)}^{\frac{1}{2}}}{\beta e^{2}}=\frac{\alpha e-\beta ce-4G}{\beta e^{2}}$$

Let

$$h^{M}\left(k,t\right)=t+k+2\sqrt{2}\frac{{\left(\beta e^{2}Gk+\beta Ce^{2}-\alpha eG+\beta ceG+2G^{2}\right)}^{\frac{1}{2}}}{\beta e^{2}}-\frac{\alpha e-\beta ce-4G}{\beta e^{2}}$$

When $\;h^{M}\left(k,t\right)<0$, F>0. The profit of the dominant HCM’s profit exceeds the opportunity cost. At this time, the HCM should choose LCT for normal production. Otherwise, as the mixed carbon policy increases, when $\;h^{M}\left(k,t\right)>0$, F<0. The dominant HCM’s profit is less than the opportunity cost and it will stop production. In the same way, the boundary condition when the subordinate HCM’s profit is equal to the opportunity cost is

$$t+k+\frac{4{\left(\beta e^{2}Gk+\beta Ce^{2}-\alpha eG+\beta ceG+4G^{2}\right)}^{\frac{1}{2}}}{\beta e^{2}}=\frac{\alpha e-\beta cek-8G}{\beta e^{2}}$$

Let

$$h^{R}\left(k,t\right)=t+k+\frac{4{\left(\beta e^{2}Gk+\beta Ce^{2}-\alpha eG+\beta ceG+4G^{2}\right)}^{\frac{1}{2}}}{\beta e^{2}}-\frac{\alpha e-\beta ce-8G}{\beta e^{2}}$$

When $h^{R}\left(k,t\right)<0$, F>0. The subordinate HCM’s profit exceeds the opportunity cost. At this time, the HCM should choose LCT for normal production. Otherwise, as the mixed carbon policy increases, when $h^{R}\left(k,t\right)>0$, F<0. The subordinate HCM’s profit is less than the opportunity cost and it will stop production. □
